# Intramolecular ^13^C analysis of tree rings provides multiple plant ecophysiology signals covering decades

**DOI:** 10.1038/s41598-018-23422-2

**Published:** 2018-03-22

**Authors:** Thomas Wieloch, Ina Ehlers, Jun Yu, David Frank, Michael Grabner, Arthur Gessler, Jürgen Schleucher

**Affiliations:** 1 Department of Medical Biochemistry and Biophysics, Umeå University, 90187 Umeå, Sweden; 2 Department of Mathematics and Mathematical Statistics, Umeå University, 90187 Umeå, Sweden; 3 Laboratory of Tree-Ring Research, University of Arizona, 85721-0045 Tucson, USA; 4 Institute of Wood Science and Technology, University of Natural Resources and Life Sciences Vienna, 3430 Tulln an der Donau, Austria; 5 Forest Dynamics, Swiss Federal Research Institute WSL, 8903 Birmensdorf, Switzerland; 6 Institute of Terrestrial Ecosystems, ETH Zurich, 8092 Zürich, Switzerland

## Abstract

Measurements of carbon isotope contents of plant organic matter provide important information in diverse fields such as plant breeding, ecophysiology, biogeochemistry and paleoclimatology. They are currently based on ^13^C/^12^C ratios of specific, whole metabolites, but we show here that intramolecular ratios provide higher resolution information. In the glucose units of tree-ring cellulose of 12 tree species, we detected large differences in ^13^C/^12^C ratios (>10‰) among carbon atoms, which provide isotopically distinct inputs to major global C pools, including wood and soil organic matter. Thus, considering position-specific differences can improve characterisation of soil-to-atmosphere carbon fluxes and soil metabolism. In a *Pinus nigra* tree-ring archive formed from 1961 to 1995, we found novel ^13^C signals, and show that intramolecular analysis enables more comprehensive and precise signal extraction from tree rings, and thus higher resolution reconstruction of plants’ responses to climate change. Moreover, we propose an ecophysiological mechanism for the introduction of a ^13^C signal, which links an environmental shift to the triggered metabolic shift and its intramolecular ^13^C signature. In conclusion, intramolecular ^13^C analyses can provide valuable new information about long-term metabolic dynamics for numerous applications.

## Introduction

In-depth understanding of the earth system is required to preserve intact ecosystems and protect biodiversity, maintain food supplies and secure other resources in the context of ongoing environmental change. Measurements of stable carbon isotope ratios (^13^C/^12^C ratios, expressed as δ^13^C) have helped to develop such understanding by (*inter alia*) constraining global C cycle models^1^ and illuminating plant-environment interactions^2^. However, there are major uncertainties in earth system models due to incomplete characterisation of soil microbial, biogeochemical, plant physiological, and climatic processes. Notably, estimation of soil-to-atmosphere CO_2_ fluxes based on δ^13^C analysis is impeded by (*inter alia*) lack of knowledge about ^13^C fractionations by soil microbes^3^. Similarly, simulated C exchange fluxes between the atmosphere and biosphere are insufficiently constrained due to limited understanding of CO_2_ fertilization effects^4^, i.e., the increase in plant carbon sequestration associated with rising atmospheric [CO_2_].

Natural plant archives, including tree rings, enable ^13^C analyses over decadal to millennial time scales. This is important because covering such timeframes by direct monitoring or manipulative experiments is impossible, but it is essential for robustly constraining vegetation modules of earth system models and predicting changes in plant productivity under climate change. However, the information that can be extracted from archives is currently limited by lack of sufficient understanding of plant ^13^C fractionation. There are well-established differences in ^13^C abundances among intramolecular C positions in various metabolites, including glucose^5–9^, but extant studies in plant ecophysiology and the earth sciences report conventional ^13^C/^12^C measurements of whole molecules. These whole-molecule studies rely on the assumption that intramolecular variability is negligible. Here, we test this assumption and investigate the potential of intramolecular ^13^C measurements for extracting information from archives.

To analyse effects of intramolecular ^13^C variation, we distinguish two major ^13^C fractionation systems, diffusion-Rubisco (DR) fractionation and post-Rubisco (PR) fractionation. DR fractionation refers to the ^13^C fractionation by CO_2_ diffusion from ambient air into plant chloroplasts and Rubisco-mediated CO_2_ fixation^2^, previously called photosynthetic fractionation^10^. The rationale for the change in nomenclature is outlined below. Rubisco adds a single carbon from CO_2_ to ribulose-1,5-bisphosphate. Therefore, DR fractionation cannot cause intramolecular ^13^C variation, i.e. it is not position-specific. In contrast, PR fractionation denotes ^13^C fractionation by enzymes acting downstream of Rubisco. This type of fractionation is known to occur at individual C positions within metabolites^11^, i.e. it is position-specific. PR fractionation occurs at metabolic branch points^9^. Theoretically, events such as changes in metabolite allocation at an isotope-sensitive branch point will change the intramolecular ^13^C pattern. Thus, intramolecular ^13^C distributions should carry signals reflecting such shifts in metabolic branching.

To explore the potential of the intramolecular level, we measured intramolecular ^13^C distributions in the glucose units of tree-ring cellulose of an annually resolved *Pinus nigra* tree-ring series. The samples originate from a moisture limited site, and cover the period 1961–1995. We then conducted a comparative time-series analysis with conventional whole-molecule and intramolecular ^13^C/^12^C ratios. Furthermore, we measured the same distributions in samples of six angiosperm and five additional gymnosperm species from globally distributed sites.

We report six findings. First, ^13^C distributions show intramolecular differences of the order of 10‰. Second, while a signal due to DR fractionation is present at some C positions of *Pinus nigra* tree-ring glucose, it is attenuated or even absent at other positions. Third, the intramolecular approach enables better description and prediction of environmental variables. Fourth, Hierarchical Cluster Analysis revealed PR signals at several C positions. Fifth, environmental drivers control PR fractionation. Finally, we propose an ecophysiological mechanism for the origin of a PR signal linking an environmental shift with a defined metabolic shift, which leaves its isotopic signature in the tree-ring archive. We conclude that intramolecular ^13^C analysis greatly extends the information that can be extracted from tree-ring archives.

## Intramolecular ^13^C fractionation: Concepts and nomenclature

As described above, we distinguish here between diffusion-Rubisco (DR) and post-Rubisco (PR) fractionation. Synonyms for DR and PR fractionation are photosynthetic fractionation, and post-photosynthetic or post-carboxylation fractionation, respectively^10,12^. Photosynthesis involves the action of several fractionating enzymes, e.g. Rubisco, transketolase, and aldolase^13^, but the term photosynthetic fractionation usually refers exclusively to fractionation by CO_2_ diffusion and Rubisco-catalysed carboxylation. Fractionations occurring downstream of Rubisco carboxylation have been called post-carboxylation fractionation, but other fractionating carboxylases occur in plants. Robust understanding of high-resolution intramolecular ^13^C fractionation requires unambiguous terminology. Therefore, we here introduce the terms DR and PR fractionation, which allow the classification of plant ^13^C fractionations into non-position-specific and position-specific processes.

**Figure 1:**
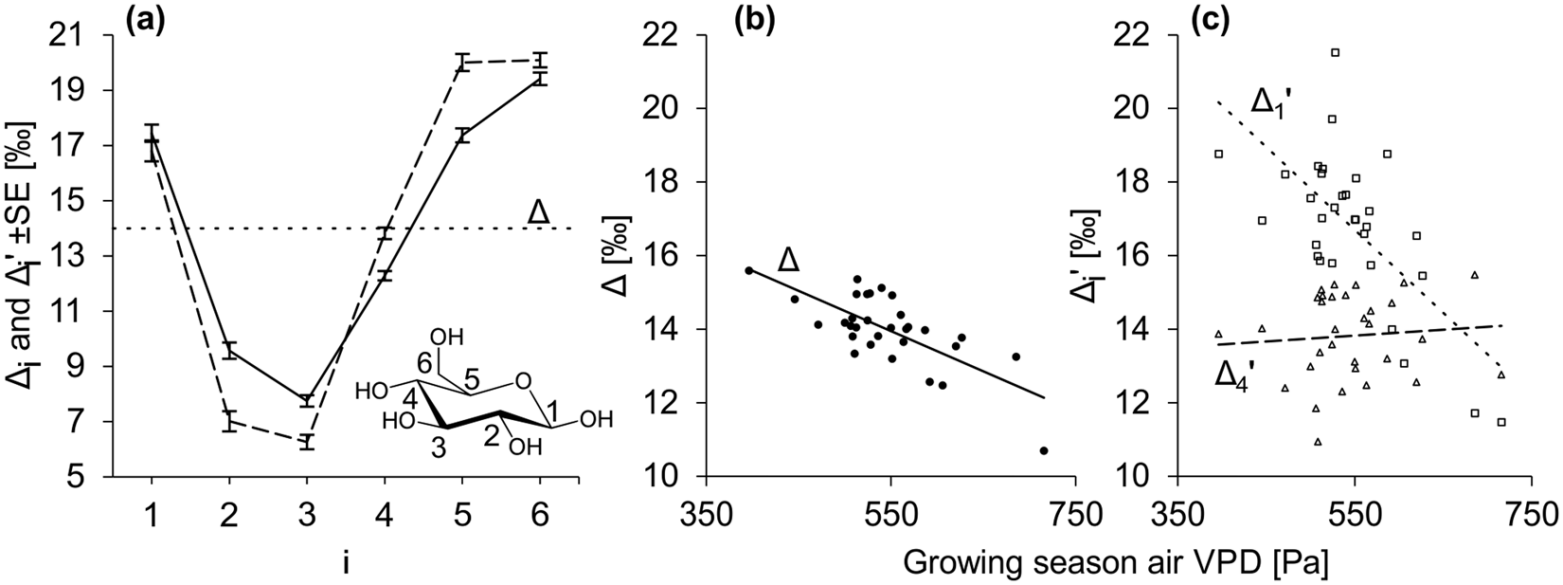
Intramolecular ^13^C distributions and effects of growing season air vapour pressure deficit (VPD) on ^13^C discrimination. Data were acquired for tree-ring glucose of *Pinus nigra* laid down from 1961 to 1995 at a site in the Vienna basin. (**a**) Intramolecular ^13^C distributions (means over 31 years) expressed in terms of intramolecular ^13^C discrimination. Solid line, observed distribution (Δ_i_); dashed line, TPC-free distribution (Δ_i_′); dotted line, hypothetical distribution without positional ^13^C effects. Insert: Glucose unit of cellulose showing intramolecular locations of carbon positions, i. (**b**,**c**) Effects of VPD on whole-molecule ^13^C discrimination, Δ and on positional ^13^C discrimination at C-1 and C-4; Δ_1_′ and Δ_4_′, respectively. Linear regression demonstrates highly significant negative relationships between VPD and both Δ and Δ_1_′, and no detectable relationship between VPD and Δ_4_′ (ordinary least squares regressions, n = 31, Δ = −0.011VPD + 20.0, r = −0.72, p = 5.4*10^−6^; Δ_1_′ = −0.023VPD + 29.1, r = −0.68, p = 3*10^−5^; Δ_4_′ = 0.002VPD + 12.9, r = 0.09, p = 0.64).

^13^C discrimination, a measure of the ^13^C fractionation in plants, has been defined^14^ as:

1 $${\rm{\Delta }}=({{\rm{R}}}_{{\rm{a}}}{/{\rm{R}}}_{{\rm{p}}})-1$$

where R_a_ and R_p_ are the ^13^C/^12^C ratios of a carbon source and plant sample, respectively. To screen for intramolecular ^13^C signals, suitable isotope parameters are required. In analogy to Δ, we define positional ^13^C discrimination as:

2 $${{\rm{\Delta }}}_{{\rm{i}}}=({{\rm{R}}}_{{\rm{a}}}{/{\rm{R}}}_{{\rm{pi}}})-1$$

where R_pi_ is the ^13^C/^12^C ratio at carbon position i of a plant metabolite (see Fig. 1a for carbon assignments). With R_a_ and R_pi_ expressed in terms of the conventional δ scale as δ^13^C_a_ and δ^13^C_pi_, respectively, Δ_i_ is given as:

3 $${{\rm{\Delta }}}_{{\rm{i}}}=({{\rm{\delta }}}^{13}{{\rm{C}}}_{{\rm{a}}}{-{\rm{\delta }}}^{13}{{\rm{C}}}_{{\rm{pi}}})/({1+{\rm{\delta }}}^{13}{{\rm{C}}}_{{\rm{pi}}})$$

A process known as triose phosphate cycling (TPC) involves scrambling of substantial proportions (20–25%) of carbon between symmetry-related carbon positions in tree-ring glucose and can potentially confound existing intramolecular ^13^C signals, particularly leaf-level signals. Below, we present a convenient method for removing the effect of TPC from observed intramolecular ^13^C distributions of hexoses and verify its suitability. TPC-free positional ^13^C discrimination, Δ_i_′, is then given as:

4 $${{\rm{\Delta }}}_{{\rm{i}}}^{\prime} =({{\rm{R}}}_{{\rm{a}}}{/{\rm{R}}}_{{\rm{pi}}}^{\prime} )-1$$

and, in terms of δ, as:

5 $${{\rm{\Delta }}}_{{\rm{i}}}^{\prime} =({{\rm{\delta }}}^{13}{{\rm{C}}}_{{\rm{a}}}{-{\rm{\delta }}}^{13}{{\rm{C}}}_{{\rm{pi}}}^{\prime} )/{(1+{\rm{\delta }}}^{13}{{\rm{C}}}_{{\rm{pi}}}^{\prime} )$$

where isotope parameters marked by a prime are free of TPC-related variation. Δ_i_ and Δ_i_′ each have specific uses: Δ_i_, denoting observed ^13^C abundances, is relevant when tree-ring glucose enters microbiological and biogeochemical processes; Δ_i_′, denoting TPC-free ^13^C abundances, enables better understanding of ^13^C fractionation systems in plants.

## Results

### Tree-ring glucose exhibits a non-random intramolecular ^13^C distribution

First, we examined intramolecular ^13^C distributions by averaging all 31 annual distributions of the raw and TPC-free datasets (Δ_i_ and Δ_i_′, respectively) of *Pinus nigra*. Both distributions show non-random patterns with intramolecular differences exceeding 10‰ (Fig. 1a; solid and dashed lines, respectively). Positional differences are more pronounced in Δ_i_′ than in Δ_i_. This is as expected, given that Δ_i_′ is free of the influence of TPC, which causes partial averaging of positional ^13^C abundances (see below). We obtained similar intramolecular ^13^C distributions for six angiosperm and five additional gymnosperm species from different sites with global coverage (Fig. S1, Table S1). Our observations of distinct ^13^C patterns in tree-ring glucose are consistent with observations of glucose derived from other metabolites^15–18^. As mentioned above, DR fractionation cannot induce intramolecular ^13^C differences. Thus, observed patterns show that PR fractionations have clearly detectable effects.

### The observable DR signal in tree-ring glucose is position-specific

Above, we show that tree-ring glucose exhibits a pronounced intramolecular ^13^C pattern, which can be attributed to PR fractionation effects. If this pattern varies over time, then intramolecular ^13^C abundances may carry unique information about long-term metabolic dynamics. Therefore, all subsequent analyses focus on properties related to temporal variability of the intramolecular ^13^C patterns (i.e. intramolecular ^13^C signals).

A tree ring formed in a particular year may have had significant input of stored glucose monomers from previous years. If so, ^13^C time series would exhibit autocorrelation signals. Therefore, we tested all ^13^C time series (Δ, Δ_i_, Δ_i_′) for autocorrelation, applying temporal lags of one to three years (see SI). We found no evidence of autocorrelation, showing that interannual carryover of signals is negligible. Thus, all subsequent analyses focused on conditions during the year of tree-ring formation.

DR fractionation may be affected by diverse environmental variables^19^. It is routinely evaluated by measurements of whole-molecule ^13^C discrimination, Δ^20^. An underlying assumption is that DR fractionation controls Δ. To search for the most influential environmental variable, we correlated Δ with air vapour pressure deficit, precipitation, soil moisture, air temperature, and global radiation during the growing season (VPD, PRE, SM, TMP, RAD, respectively; the method used to estimate the growing season is described in SI). VPD was found to be most strongly correlated with Δ (VPD, r = −0.72, p = 5*10^−6^; PRE, r = 0.44, p = 0.013; SM, r = 0.38, p = 0.038; TMP, r = −0.38, p = 0.033; RAD, r = −0.58, p = 7*10^−4^; n = 31). The strong negative VPD dependency is consistent with expectations for a moisture-limited site, published relationships and the well-established mechanisms underlying DR fractionation^2,19^. Thus, the variability of DR fractionation is reflected by the variability of VPD in the first approximation. This establishes VPD as a proxy of DR fractionation under given conditions.

If DR fractionation was the only temporally variable fractionation process in plants, its signal strength should be equal at all positional time series of ^13^C discrimination, Δ_i_′ (see above). We tested this by analysing the linear relationships between Δ_i_′ and VPD. We found that VPD signal strengths vary among Δ_i_′ (Fig. S3). The largest deviations from uniformity were detected in Δ_1_′ and Δ_4_′ (Figs. 1b,c and S3). While the slope of the Δ_1_′~VPD regression is significantly steeper than the slope of the Δ~VPD regression (p = 0.02, see ANCOVA results in Table S4), the slope of the Δ_4_′~VPD regression is not significantly different from zero (p = 0.64). Thus, the VPD signal is stronger in Δ_1_′ than in Δ, and undetectable in Δ_4_′, which implies that the DR signal is transmitted into tree-ring glucose in a position-specific manner.

### The intramolecular approach enables better description and prediction of environmental variables

Correlation coefficients for the Δ~VPD and Δ_1_′~VPD relationships are similar (r = −0.72 and −0.68, respectively). Thus, simple linear regression modelling provided no indications that Δ_i_′ is superior to Δ as a proxy of environmental variables. Therefore, we tested the feasibility of capturing a higher-quality VPD signal using Δ_i_′ in a more sophisticated modelling approach. Combining multiple linear regression modelling with automatic model selection, we generated a Δ_i_′ model that describes VPD more precisely than the corresponding Δ model (VPD~Δ_1_′ + Δ_3_′ + Δ_5_′, adjR^2^ = 0.60, p = 4*10^−6^ vs. VPD~Δ, adjR^2^ = 0.50, p = 5.4*10^−6^). In contrast to R^2^, model evaluation by adjR^2^ takes the number of explanatory variables into account, enabling comparison of models with different numbers of explanatory variables. Next, we tested the predictive abilities of both models by 10-fold cross-validation. We found that the Δ_i_′ model predicts VPD more precisely (Q^2^ = 0.52 vs. Q^2^ = 0.43, where Q^2^ denotes the cross-validated R^2^). These findings show that the intramolecular approach enables more precise description and prediction of VPD, and suggests that Δ_i_′ might allow for improved climate reconstructions.

### Tree-ring glucose contains several distinct intramolecular ^13^C signals

**Figure 2:**
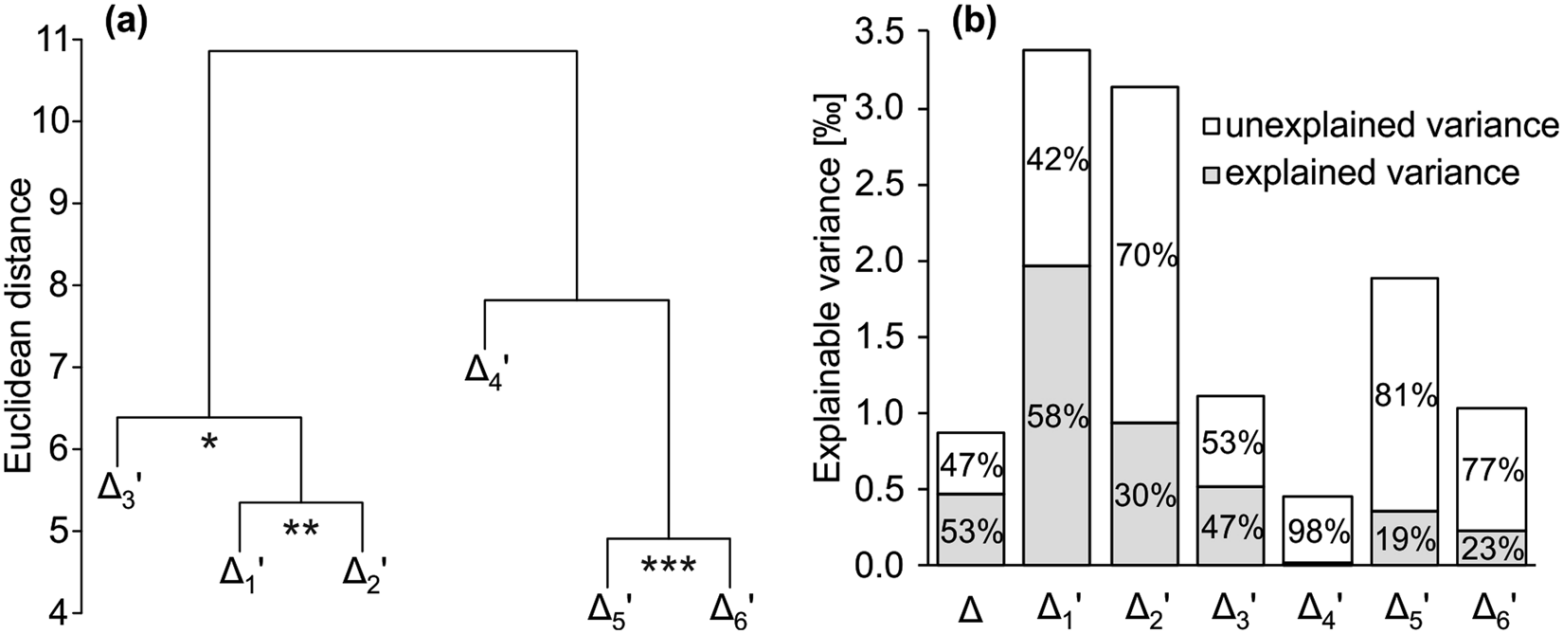
Common variability among and components of variance in time-series of ^13^C discrimination. Data were acquired for tree-ring glucose of *Pinus nigra* laid down from 1961 to 1995 at a site in the Vienna basin. (**a**) Dendrogram showing clustering of time series of the TPC-free intramolecular ^13^C discrimination, Δ_i_′. Asterisks denote the significance of correlation between Δ_i_′ forming a cluster (*p ≤ 0.05; **p ≤ 10^−2^; ***p ≤ 10^−3^, n = 31). (**b**) De-convolution of the explainable component of variance in Δ, and Δ_i_′ into an explained and an unexplained component of variance according to previous authors^21^. Explainable variance denotes the total variance minus estimated error variance. Explained variance denotes the component of variance accounted for by growing season air vapour pressure deficit. Unexplained varaince denotes the component of variance not accounted for by independent variables.

Due to the single carbon addition by Rubisco, DR fractionation equally affects all carbon entering photosynthesis (see above). However, the results presented above show that the DR signal is not equally distributed over all carbon positions of the downstream metabolite tree-ring glucose (Figs. 1b,c and S3), suggesting that PR fractionations influence Δ_i_′, and have had varying effects in the 31-year tree-ring series. To confirm this implication, we screened for position-specific signals by hierarchical cluster analysis of Δ_i_′. We found four clusters: Δ_1_′ to Δ_2_′, Δ_3_′, Δ_4_′, and Δ_5_′ to Δ_6_′ (Fig. 2a). Cluster formation and separation occur due to common and distinct variability, respectively. For instance, Δ_1_′ and Δ_2_′ as well as Δ_5_′ and Δ_6_′ share significantly correlated common signals (r = 0.54, p = 1.65*10^−3^, and r = 0.61, p = 2.36*10^−4^, respectively, n = 31). As Δ_1_′ and Δ_6_′ as well as Δ_2_′ and Δ_5_′ are uncorrelated (r = 0.08, p = 0.68, and r = 0.11, p = 0.71, respectively, n = 31), detected common signals are independent of each other. Thus, PR fractionations introduce ^13^C signals on top of the DR fractionation signal. Moreover, independence among clusters implies that intramolecular ^13^C patterns of tree-ring glucose vary on interannual timescales.

### Ecophysiological information is better resolved on the intramolecular level

Observation of multiple intramolecular ^13^C signals implies that Δ is a composite of several signals with distinct physiological origins, and raises questions about the relative importance of DR and PR fractionation for Δ and Δ_i_′. To address these questions, we first estimated the error variances in Δ and Δ_i_′, which reflect random components of variance caused by finite measurement precision, which differs strongly between Δ and Δ_i_′. Then, we calculated explainable components of variance, which may theoretically be linked to specific ecophysiological processes through modelling^21^. We then de-convoluted the explainable variance into a component explained by growing season air VPD and an unexplained component. With VPD as a proxy of DR fractionation (see above), this approach enables estimation of the relative importance of DR versus PR fractionation.

The explainable variance differs substantially among Δ_i_′, from 0.45‰ for Δ_4_′ to 3.37‰ for Δ_1_′ (Fig. 2b). High values indicate substantial fractionation effects. From this perspective, Δ_1_′, Δ_2_′, Δ_5_′ have high potential, and Δ_4_′ has low potential for extracting ecophysiological information. In most Δ_i_′, the unexplained component of variance exceeds the explained component. In Δ, both components of variance are similar. These findings suggest that PR fractionation has non-negligible effects on Δ and all Δ_i_′. Moreover, they emphasise the generally high potential for extracting multiple ecophysiological signals from intramolecular-level ^13^C data, particularly novel signals reflecting dynamic regulation of enzyme reactions downstream of Rubisco.

## Discussion

Intramolecular ^13^C distributions of tree-ring glucose are generally non-random (Fig. 1a and S1). This finding is consistent with previous observations of glucose derived from other species, tissues, and metabolites^15–18^. Detected intramolecular ^13^C differences exceed 10‰. Thus, they are an order of magnitude larger than intra-annual ^13^C variations of atmospheric CO_2_^22^. Moreover, their magnitude is similar to ^13^C differences reported for distinct plant metabolites^23^, and to the whole ^13^C range reported for bulk plant materials, including C3 and C4 plants^24^.

Wood cellulose (composed of glucose units) is one of the largest global C pools^25^ and thus may strongly influence responses of the global C cycle to climatic changes. More specifically, wood cellulose is a major contributor to soil organic matter and, hence, subject to numerous biogeochemical transformations. These transformations are incompletely understood with respect to contributions of different microbial communities, turnover times of soil organic matter components, and responses to climatic changes^26^.

Isotopes are powerful tools for analysing soil C turnover and associated phenomena. However, their use requires information about both fractionation effects of microbial communities^3^ and the isotopic composition of soil substrates. For instance, soil cellulose decomposition occurs under both aerobic and anaerobic conditions via several metabolic pathways^27^. Because of the non-random ^13^C distribution of wood glucose (Fig. 1a, solid line and Fig. S1), different breakdown pathways will liberate CO_2_ with distinct δ^13^C fingerprints. The δ^13^C of liberated CO_2_ will equal the δ^13^C of substrate glucose, if glucose molecules are completely respired. If glucose is fermented (liberating C-3 and C-4), CO_2_ with approximately 2.5‰ more positive δ^13^C values will be released (Fig. S1). Although this reasoning neglects fractionation effects of decarboxylation reactions, it illustrates the association of distinct breakdown pathways with substantial ^13^C differences in respired CO_2_. Thus, it shows that considering positional ^13^C differences in soil organic matter will enable better characterisation of C turnover pathways and quantification of heterotrophic soil respiration. This, in turn, will help reduce uncertainties in regional- to global-scale models of terrestrial productivity, and earth system models^28^.

Our data provide the first proof of temporal variability in intramolecular ^13^C patterns; more specifically, interannual variation in the ^13^C patterns of glucose derived from *Pinus nigra* tree rings (Fig. 2a). As non-random intramolecular ^13^C patterns result from specific isotopic effects of enzymes acting downstream of Rubisco^29^, these observations establish a clear link between ^13^C abundances of plant organic matter and temporal variability in metabolic dynamics.

Our analyses show that intramolecular ^13^C abundances of tree-ring glucose contain information about the dynamics of both primary CO_2_ fixation and downstream metabolic processes. While DR fractionation explains much of the interannual variability of Δ, PR fractionations are clearly not negligible (Figs. 2a,b). This may explain why the sensitivity of whole-molecule δ^13^C values in tree rings to ecophysiological parameters is highly variable^30^, and why coefficients of determination (R^2^) obtained by attempts to model Δ rarely exceed 50%. This, in turn, suggests that multiple intramolecular signals are generally present in ^13^C datasets, and that intramolecular ^13^C analysis offers considerable scope to improve the resolution and robustness of ^13^C analyses.

While the mechanisms behind observed PR fractionation signals require further attention, intramolecular ^13^C ratios clearly offer more information than whole-molecule ratios (Figs. 2a,b). This will likely facilitate retrospective assessment of plant ecophysiological and environmental traits unrelated to the diffusion-Rubisco mechanism. To illustrate this point, we relate the magnitudes of observed Δ_i_′~VPD dependencies to published magnitudes of enzyme isotope effects, and derive a hypothesis for the physiological origin of PR fractionations at glucose C-1 and C-2.

Δ_1_′ and Δ_2_′ exhibit higher degrees of explainable variance than any other Δ_i_′, and are highly correlated with each other (Figs. 2a,b). In comparison, the correlation between Δ_3_′ and the average over Δ_1_′ and Δ_2_′ is less significant. Above, we established VPD as proxy of DR fractionation under given conditions, and we found significant VPD correlations with Δ_1_′ (r = −0.68, p = 3*10^−5^), Δ_2_′ (r = −0.49, p = 5.5*10^−3^) and Δ_3_′ (r = −0.51, p = 3.5*10^−3^). However, as shown in Figure S3, regression slopes between VPD and Δ_i_′ decline in the order Δ_1_′ (b_1_′ = −0.0226 ± 0.0046SE ‰ Pa^−1^), Δ_2_′ (b_2_′ = −0.0156 ± 0.0052SE ‰ Pa^−1^) and Δ_3_′ (b_3_′ = −0.0116 ± 0.0037SE ‰ Pa^−1^). DR fractionation is not position-specific, and can therefore only introduce regression slopes of equal size. Significant VPD correlations suggest that the DR signal is present at Δ_1_′ to Δ_3_′. Above-average explainable variance, a strong common signal, and steeper VPD slopes indicate that Δ_1_′ and Δ_2_′ contain additional VPD-dependent PR signals. Thus, assuming that b_3_′ represents the common DR signal, the PR contributions to the Δ_1_′~VPD and Δ_2_′~VPD slopes are b_1PR_′ = b_1_′–b_3_′ and b_2PR_′ = b_2_′–b_3_′, respectively.

Phosphoglucose isomerase (PGI, EC 5.3.1.9) catalyses conversion of fructose-6-phosphate to glucose-6-phosphate (G6P), which is used in formation of starch or tree-ring cellulose. It is the only enzyme that simultaneously modifies C-1 and C-2 bonds of G6P and hence glucose units in tree-ring cellulose, and can therefore introduce isotope effects of substantial size at these positions (primary isotope effects). Hence, PGI is the most likely generator of the correlated PR signals in Δ_1_′ and Δ_2_′. Bacterial glucose isomerase (EC 5.3.1.5) has substantial equilibrium and kinetic isotope effects at glucose C-1 and C-2 (EIE_C-1_ = −13‰, EIE_C-2_ = 7‰, KIE_C-1_ = 5‰, KIE_C-2_ = 15‰), according to previous authors^18^, who hypothesis that plant PGI should have similar effects, as it operates by the same reaction mechanism. Hence, shifts of the PGI reaction from irreversible to equilibrium conditions will be accompanied by correlated ^13^C shifts at C-1 and C-2 of the reaction product, G6P. Magnitudes of these ^13^C shifts are proportional to the differences between corresponding kinetic and equilibrium isotope effects, and ^13^C shifts at C-1 and C-2 of G6P are linearly related as (KIE_C-1_-EIE_C-1_)/(KIE_C-2_-EIE_C-2_) = 2.25. That is, a given shift at G6P C-2 will be accompanied by a 2.25-fold larger shift at C-1. This ratio should equal the ratio of the PR contributions to the Δ_1_′~VPD and Δ_2_′~VPD regression slopes, b_1PR_′/b_2PR_′. We found that b_1PR_′/b_2PR_′ = 2.74 (+1.35SE, −0.60SE), which is consistent with a PGI-related mechanism introducing a ^13^C signal in Δ_1_′ and Δ_2_′.

From an ecophysiological perspective, the occurrence of PGI-driven fractionation is plausible for the following reasons. In isohydric plants like *Pinus nigra*, strong negative relationships between VPD and both stomatal conductance and intercellular [CO_2_] can be expected^31^. At high intercellular [CO_2_], plants photosynthesise at high rates, and stromal PGI is strongly displaced from equilibrium^32–34^. As intercellular [CO_2_] declines, plants photosynthesise at lower rates, and stromal PGI shifts towards equilibrium^32^. According to published isotope effects^18^, a shift towards equilibrium results in ^13^C enrichments at C-1 and C-2 of stromal G6P. From G6P, the signal is transmitted to transitory starch and the glucose units of tree-ring cellulose derived therefrom. Low intercellular [CO_2_], as induced by stomatal closure due to high VPD, is associated with ^13^C enrichment by the DR fractionation system. Consequently, DR and PR fractionation at C-1 and C-2 have synergistic effects, and lead to steeper Δ_1_′ ~ VPD and Δ_2_′ ~ VPD regression slopes. A regulated shift towards PGI equilibrium may putatively facilitate stabilisation of the Calvin-Benson cycle^35^, which is probably most important when intercellular [CO_2_] is low. Thus, a PGI-related mechanism can explain enhanced Δ_1_′ and Δ_2_′ fractionations and is ecophysiologically plausible.

Analysis of intramolecular variation in isotope ratios is intended to resolve multiple ecophysiological signals using several information channels. In that sense, it is conceptually related to the so-called “dual-isotope approach”; the independent, but simultaneous, examination of stomatal conductance and carbon assimilation through combined analysis of whole-molecule δ^13^C and δ^18^O of plant organic matter^36^. In its current form, however, application of such dual-isotope analysis depends on several assumptions, which impedes its widespread implementation^37^. One problem noted by the cited authors is that stomatal conductance and carbon assimilation are not the only processes that modulate isotope ratios. Our observation of PR fractionation, which the dual-isotope concept neglects, highlights this challenge.

The sensitivity of Δ to multiple ecophysiological variables (Figs. 2a,b) hinders attempts to model the ^13^C fractionation system of plants and to derive ecophysiological and environmental information from Δ measurements. Generally, deconvolution of several signals with only one observable variable is not feasible. In contrast, resolution of six partly independent intramolecular ^13^C variables (Fig. 2a) offers a conceptual shift from underdetermined towards fully or even overdetermined model systems. This development can potentially reduce numbers of confounding factors and (hence) model uncertainty. The most powerful approaches may combine intramolecular and multi-isotope techniques, which would offer the highest number of independent isotope information channels. In future, estimations of physiological and environmental parameters including source isotope compositions will most likely rely on such “multichannel” approaches.

Intrinsic water-use efficiency (iWUE) is defined as the ratio between the rates of carbon assimilation and transpiration. It is a major determinant of plant performance at water-limited sites^2^. DR fractionation is correlated with iWUE, and Δ is often used as proxy of iWUE^19^. Our results indicate that a purer DR signal can be obtained on the level of intramolecular ^13^C abundances. Thus, models based on Δ_i_′ may provide better estimates of iWUE.

We found that a statistical model of VPD based on Δ_i_′ has greater descriptive and predictive capacities than the corresponding Δ model. This finding is especially noteworthy given the lower achievable accuracy of Δ_i_′ measurements compared to Δ measurements (SD ± 1‰ vs. SD ± 0.1‰, respectively). Currently, Δ_i_′ measurements are time consuming and thus limited to small sample sets. We expect that Δ_i_′ applications will improve markedly with anticipated analytical advancements and with the further elucidation of PR fractionation effects, which might allow more sophisticated mechanistic modelling.

Intramolecular ^13^C abundances are functions of environmental and related physiological variables, studied here at annual resolution. The approach is generally suitable for analysis of samples covering much longer timeframes^38^, far exceeding the scope of manipulation experiments or direct observation. However, upscaling to these timeframes requires an assessment of the temporal robustness of ^13^C signals. In nature, wood cellulose often persists for long periods, and is datable with high accuracy. Several tree-ring chronologies with annual resolution and calendric exactness encompass the entire Holocene^39^. Subfossil wood samples date back to the last interglacial period, ≈130,000 to 115,000 BP^40,41^. Thus, intramolecular ^13^C distributions in wood are promising archives of information about physiological and environmental conditions in past decades, centuries, and millennia. Position-specific isotope abundances may be particularly valuable for acquiring information (which is difficult to acquire by any other available technology) about the capacity of different plant species to acclimatise and adapt to long-term environmental changes. This, in turn, might aid attempts to identify suitable plants, cultivars and genotypes for changing environments.

We anticipate that intramolecular ^13^C measurements will complement whole-molecule stable isotope measurements and multi-isotope approaches in several applications. These include: prediction of ^13^C abundances of CO_2_ formed by different respiratory pathways; characterisation of the C metabolism of soil microbial communities; analyses of soil carbon turnover; elucidation of plants’ physiological responses to environmental changes and their long-term acclimatisation (in periods and conditions covered by calibrating data); and reconstructions of plant physiological and environmental traits based on mechanistic models (outside periods and conditions covered by calibrating data).

## Methods

Additional information is provided under Supporting Information.

### Site and samples

We used samples of annual rings of 19 *Pinus nigra* Arnold trees (two cores per tree) from the Bierhäuselberg site (Vienna region, Austria), which has shallow, very dry soil. Both the site and samples have been previously described in detail^42^. In addition, we used dated tree-ring samples, pooling 5–10 annual rings of 11 angiosperm and gymnosperm species from ecologically different sites with global coverage (Table S1).

### Sample preparation

We carefully separated dated *Pinus nigra* tree rings (from 1961 to 1995) using a binocular microscope and a scalpel, and combined rings in annual pools. Thus, our data represent properties of the tree species at the site rather than individual trees. Pooled samples were ground (Retsch® MM400, Haan, Germany) and their glucose contents were converted into 1,2-O-isopropylidene-α-D-glucofuranose following a published protocol^43^. Samples of 11 additional angiosperm and gymnosperm species were processed in the same way, but in a final step their glucose contents were converted into 3,6-anhydro-1,2-O-isopropylidene-α-D-glucofuranose^43^. Checks by ^1^H NMR showed that sample purity was ≥99.9%.

### ^13^C EA-IRMS and ^13^C NMR spectroscopy

Conventional δ^13^C_VPDB_ measurements of the glucose derivative were acquired for *Pinus nigra* samples. Quantitative 1D ^13^C NMR spectra were collected^44^ using a Bruker 400 MHz AVANCE III instrument equipped with a 5 mm BBFO SmartProbe^TM^ (Bruker BioSpin GmbH, Rheinstetten, Germany). We recorded and processed 30 spectra per *Pinus nigra* sample and eight spectra per sample of the additional species using TopSpin^TM^ 3.1 (Bruker BioSpin GmbH, Rheinstetten, Germany). We excluded *Pinus nigra* samples from 1977, 1978, 1981, and 1982 because they were too small.

### Calculation of Δ_i_ and Δ_i_′

Integration of ^13^C NMR spectra resulted in average signal integrals, S_i_, of specific carbon positions of the glucose derivatives, i = {C-1,…, C-6, C-q, C-Me1, C-Me2}. Each carbon is directly bound to one or two neighbouring carbons. Calculation of ^13^C molar equivalents, S_i(c)_, considered corresponding signal satellites^45^. Removal of ^13^C variation related to TPC followed methods described below, eq. (8), and resulted in TPC-free ^13^C molar equivalents, S_i(c)_′. Calculation of positional ^13^C/^12^C ratios, expressed as δ^13^C_pi_ and δ^13^C_pi_′ followed published procedures^46^. Calculation of positional discrimination, Δ_i_, and the TPC-free positional discrimination, Δ_i_′, followed eqs. (3) and (5), and incorporated reconstructed annual atmospheric δ^13^CO_2_ (=δ^13^C_a_) for the northern hemisphere^47^. As the open canopy at our site presumably allows rapid mixing of biogenic and atmospheric CO_2_, errors in Δ_i_ and Δ_i_′ due to the contribution of isotopically distinct biogenic CO_2_ should be minimal. Positional ^13^C deviations from the molecular average were calculated as Δδ^13^C_i_ = (S_i(c)_/(ΣS_i(c)_/n)−1)*10^3^ with i = {C-1, …, C-6}.

### Fractional redistribution of ^13^C signals between symmetry-related glucose carbon positions by heterotrophic triose phosphate cycling

When cellulose is synthesized, translocated sucrose is first broken down to hexoses, which are converted to UDP-glucose. During these reactions, 40 to 50% of the hexose phosphates generated are further broken down to triose phosphates, before use in cellulose synthesis. This is known as triose phosphate cycling (TPC). Triose phosphate isomerase equilibrates glyceraldehyde 3-phosphate (G3P) with dihydroxyacetone phosphate (DHAP), respectively derived from C4–6 and C1–3 portions of hexoses. Their equilibration causes carbon exchange between C1–3 and C4–6 portions of hexoses. Thus, in comparison to the hexose units of sucrose, approximately 20 to 25% of carbons in the UDP-glucose pool have been effectively redistributed between symmetry-related carbon positions, i.e. between C-1 and C-6, C-2 and C-5, C-3 and C-4. This implies that intramolecular ^13^C differences between these symmetry-related positions are partially levelled out by TPC. In the following text, we derive equations to back-calculate the intramolecular ^13^C distribution before TPC. Please note that the resulting TPC-free distribution does not represent the ^13^C distribution of any naturally occurring hexose. This is because both parts of sucrose, i.e. glucose and fructose, are used for cellulose synthesis, but differ with respect to their ^13^C distributions^8^.

### Equation for removing the averaging effect of heterotrophic TPC

With y denoting the fraction of hexose phosphates cycling through triose phosphates, and with complete triose phosphate equilibration, the fraction of carbon redistributed between symmetry-related carbon positions is given by y/2. Then, the observed ^13^C abundance at a specific hexose carbon position, C_i_, is given by:

6 $${}^{13}{\rm{C}}_{{\rm{i}}}=(1-{\rm{y}}/2){}^{13}{\rm{C}}_{{\rm{i}}}^{\prime} +({\rm{y}}/2){}^{13}{\rm{C}}_{{\rm{s}}}^{\prime} $$

and the observed ^13^C abundance of the symmetry-related carbon position, C_s_, is given by:

7 $${}^{13}{\rm{C}}_{{\rm{s}}}=(1\,-\,{\rm{y}}/2){}^{13}{\rm{C}}_{{\rm{s}}}^{\prime} +({\rm{y}}/2){}^{13}{\rm{C}}_{{\rm{i}}}^{\prime} $$

Here, ^13^C_i_′ and ^13^C_s_′ denote TPC-free ^13^C abundances. Solving eqs (6) and (7) for ^13^C_i_′, TPC-free ^13^C abundances are given by:

8 $${}^{13}{\rm{C}}_{{\rm{i}}}\text{'}=((2/{\rm{y}}-1){}^{13}{\rm{C}}_{{\rm{i}}}-{}^{13}{\rm{C}}_{{\rm{s}}})/(2/{\rm{y}}-2)$$

### Validation of the procedure

Reported estimates of proportions of carbon redistributed by TPC include 20–25% in *Quercus robur*^48^, 25% and 19% in *Quercus petraea* and *Picea abies*, respectively^49^, and 19% in various riparian tree species^50^. Thus, the fraction of carbons redistributed by TPC seems to fall within a quite narrow range in all investigated species. Both phylogenetically and in terms of wood anatomy, *Pinus nigra* is closer to *Picea abies* than to *Quercus* species. Therefore, we chose y = 0.4 as a TPC factor for calculating TPC-free ^13^C abundances (^13^C_i_′). Δ_i_ and Δ_i_′ were then calculated as described above.

As TPC averages ^13^C abundances at symmetry-related hexose positions, it should lead to correlation between symmetry-related Δ_i_ values, and these correlations should be removed by the calculation of TPC-free Δ_i_′ values. As expected for ^13^C abundances affected by TPC, Δ_i_ time series of symmetry-related glucose carbon positions correlate significantly (Table 1, values in boldface). In contrast, the TPC-free dataset, Δ_i_′, does not exhibit such a correlation pattern, indicating that co-variation introduced by TPC was removed (Table 2). In mathematical terms, TPC causes weighted averaging of carbon abundances, eqs. (6) and (7). Like any averaging, it reduces variability. Accordingly, Δ_i_′ exhibits more pronounced variation in its intramolecular distribution than Δ_i_ (Fig. 1a). Generally, averaging only has a net effect if differences are present, and the effect increases with the magnitude of the differences. This is reflected by the larger impact of removing TPC effects on Δ_2_ and Δ_5_ than on Δ_1_ and Δ_6_ (Fig. 1a).

**Table 1:** Correlation coefficients and significance levels (*p ≤ 0.05; **p ≤ 10^−2^; ***p ≤ 10^−3^; ****p ≤ 10^−4^) obtained from the Δ_i_ cross-correlation analysis (n = 31).

	Δ_1_	Δ_2_	Δ_3_	Δ_4_	Δ_5_	Δ_6_
Δ_1_	1					
Δ_2_	0.60***	1				
Δ_3_	0.31	0.52**	1			
Δ_4_	0.00	0.31	0.38*	1		
Δ_5_	0.37*	0.42*	0.24	0.39*	1	
Δ_6_	0.55**	0.48**	0.31	0.11	0.69****	1

**Table 2:** Correlation coefficients and significance levels (*p ≤ 0.05; **p ≤ 10^−2^; ***p ≤ 10^−3^) obtained from the Δ_i_′ cross-correlation analysis (n = 31).

	Δ_1_′	Δ_2_′	Δ_3_′	Δ_4_′	Δ_5_′	Δ_6_′
Δ_1_′	1					
Δ_2_′	0.54**	1				
Δ_3_′	0.31	0.48**	1			
Δ_4_′	−0.12	0.10	**−0.12**	1		
Δ_5_′	0.11	**−0.07**	0.03	0.32	1	
Δ_6_′	**0.08**	0.19	0.21	0.06	0.61***	1

### Environmental data

We acquired monthly means of precipitation, air temperature and global radiation from the Hohe Warte climate station (Central Institution for Meteorology and Geodynamics, Vienna, Austria, 16°22′ E, 48°15′ N, 203 m AMSL, WMO ID: 1103500). Deficits in air vapour pressure, VPD [Pa], were calculated following published procedures^51^. We acquired monthly means of soil moisture from a global grid dataset (CPC Soil Moisture V2, NOAA, OAR, ESRL, PSD, Boulder, Colorado, USA) for 16°15′ E, 48°15′ N. Both the climate station and the selected grid point are no more than a horizontal distance of 15 km from the sampling site with a negligible vertical offset. Thus, all data should represent site conditions well. In conifers, tracheids form over several months^52^. Thus, we calculated climate averages and sums of the growing season, which we estimated to extend from March to November (Fig. S2).

### Statistical analyses

Statistical analyses were performed in R 1.0.143. We compared regression slopes by ANCOVA using two categories and type II sum of squares. For statistical description of VPD, we first fitted the maximal model, VPD~Δ_1_′ + Δ_2_′ + Δ_3_′ + Δ_4_′ + Δ_5_′ + Δ_6_′. We arrived at the minimal adequate model by stepwise model simplification based on Akaike’s information criterion using the step() function of the Stats package with default settings. To test the predictive abilities of the simple linear regression model, VPD~Δ, and the minimal adequate model from multiple linear regression modelling, VPD~Δ_1_′ + Δ_3_′ + Δ_5_′, we performed 10-fold cross-validation using cv.lm(m = 10) and CVlm(m = 10) functions of the DAAG package. We performed Hierarchical Cluster Analysis on z-scores of Δ_i_′ using Euclidean distances and Ward’s fusion criterion for cluster formation^53^.

### Data availability

The datasets generated and analysed during the current study are available from the corresponding authors on reasonable request.

## Electronic supplementary material

Supporting Information
