# Supplementary material for: Intramolecular 13C analysis of tree rings provides multiple plant ecophysiology signals covering decades
Source: Sci Rep. 2018 Mar 22;8:5048. doi: 10.1038/s41598-018-23422-2 (PMC5864875; doi:10.1038/s41598-018-23422-2)
Supplement: Supplementary file 1 — Supporting Information [file 41598_2018_23422_MOESM1_ESM.pdf]

Intramolecular ^{13}C analysis of tree rings provides multiple plant ecophysiology signals covering decades - Supporting Information

Thomas Wieloch, Ina Ehlers, Jun Yu, David Frank, Michael Grabner, Arthur Gessler, Jürgen Schleucher

Site and samples - Additional information

Table S1 summarises spatial and temporal information for all samples included in this study. Sampling sites cover a wide range of ecological conditions. Important ecological features of the *Pinus nigra* sampling site include an "open canopy with low competition among trees", "shallow, very dry soils", and "limited or completely absent forest management disturbances"[1]. Dominant trees with umbrella-shaped crowns indicating regular water scarcity were selected for sampling. Two 5-mm cores of each of 19 trees (approximate ages, 92-156 years) were taken at breast height. These samples have been used in several former studies[1-5], for which their annual rings were carefully dated by standard dendrochronological methods[6]. To preclude growth-related signals in our data, we focused the subsampling on tree rings formed during 1961 to 1995 when all trees had reached a comparably stable canopy position. Thus, isotopic shifts due to the contribution of soil-respired CO_2 or related mechanisms should be absent or negligible.

Table S1. Spatial and temporal information for all samples included in this study.

	Species	Site	Tree rings [years]	Lat. [°]	Long. [°]	Altitude [m AMSL]
Angiosperms	Acer saccharum	Wisconsin, USA	1997-2009	45.7N	89.5W	480
	Fagus sylvatica	Austria	2005-2011	47.15N	14.28E	840
	Juglans regia	Kyrgyzstan	1850-1859	41.78N	71.93E	1400
	Liquidambar styraciflua	Tennessee, USA	2000-2005	35.9N	84.33W	230
	Quercus robur	England	1989-1994	52.83N	0.5E	25
	Shorea superba	Borneo, Malaysia	1970-1980	5.02N	117.82E	170
Gymnosperms	Juniperus zeravshanica	Uzbekistan	1850-1859	39.62N	68.49E	2200
	Phyllocladus aspleniifolius	Average of three samples:				
		Tasmania, Australia	2005-2011	43.13S	146.692E	225
		Tasmania, Australia	1980-1989	42.21S	145.43E	250
		Tasmania, Australia	1990-1999	42.21S	145.43E	250
	Picea abies	Austria	1910-1919	47.18N	14.16E	1300
	Pinus nigra	Austria	1961-1995	48.13N	16.23E	350
	Pinus ponderosa	Arizona, USA	1850-1860	32.41N	110.72W	2423
	Thuja plicata	Idaho, USA	1850-1859	47.19N	116.29W	1380

^{13}C EA-IRMS and ^{13}C NMR Spectroscopy - Additional information

For Elemental Analysis - Isotope Ratio Mass Spectrometry (EA-IRMS) measurements we used a Flash EA 2000 coupled to a DeltaV system (both from Thermo Fisher Scientific Inc., Bremen, Germany). Repeated measurements ($n \geq 2$) confirmed that the equipment provided high accuracy and precision (long-term standard deviation of instrumental measurements: ±0.15‰ SD).

For quantitative ^{13}C NMR, samples of 1,2-O-isopropylidene-α-D-glucofuranose were dissolved in 160 µl deionised water and D-acetonitrile (CAS: 2206-26-0, filling height=35 mm). Either 5 or 6 mg of the relaxation agent $Cr(acac)_3$ (CAS: 21679-31-2; 5 mg for > 80 mg samples, 6 mg for 20 to 80 mg samples) were added. Samples of 3,6-anhydro-1,2-O-isopropylidene-α-D-glucofuranose were dissolved in D-acetonitrile (filling height=35 mm), and 8 mg $Cr(acac)_3$ was added. We determined longitudinal relaxation times (T_1) by inversion recovery experiments and adjusted the recycle delay to allow for complete relaxation (≥ 10 T_1) of all relevant ^{13}C nuclei. Quantitative ^{13}C NMR spectra were acquired at 298 K using a 90° pulse of 8.07 to 8.7 µs, following published procedures[7].

By increasing the measurement time with decreasing sample amounts, we achieved signal-to-noise ratios (accumulated over all the spectra) of 900 to 1670 (average: 1270) per sample. Samples <20 mg were excluded from analysis, because too much measurement time would have been required for sufficient precision. All free induction decays were processed by the same operator and protocol: exponential apodization with a line broadening of 1 Hz; automatic phase correction plus subsequent manual phase correction if required; automatic baseline correction of the 10-130 ppm spectral range by a 3rd order polynomial. Lorentzian line shape fitting was applied for signal deconvolution. We calculated average areas under ^{13}C NMR signals, S_i, by averaging integrals over all replicate spectra.

Intramolecular ^{13}C distributions of tree-ring glucose

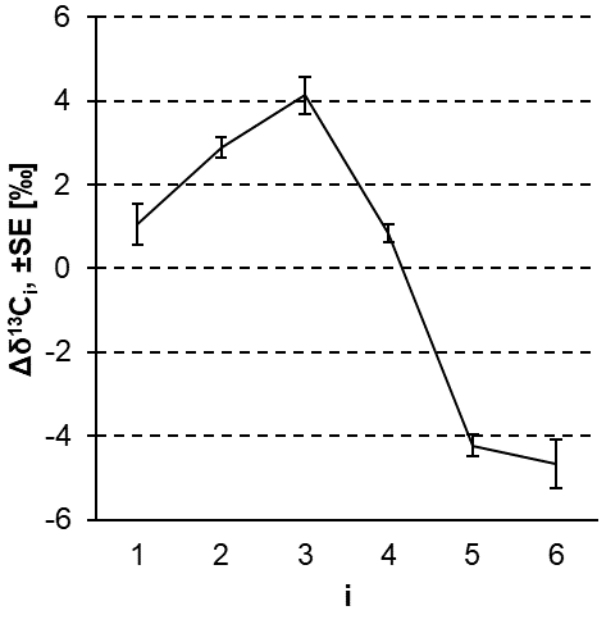

Figure S1. Intramolecular ^{13}C distributions of tree-ring glucose. Data were acquired for six angiosperm and six gymnosperm species from ecologically different sites with global coverage (Table S1). Solid line, average over all angiosperms and gymnosperms, ±SE. In the biochemical literature, intramolecular ^{13}C distributions are commonly expressed as $\Delta\delta^{13}C_i$, the deviations of positional ^{13}C abundances from the molecular average. In contrast, in the ecophysiological literature the Δ scale representing fractionation against ^{13}C is preferred. To promote comparability with previously published intramolecular ^{13}C distributions in the biochemical literature, we here express ^{13}C distributions of tree-ring glucose in terms of $\Delta\delta^{13}C_i$. Consequently, the distributions here and in the main text (Fig. 1) appear vertically mirrored around the molecular average.

Test for autocorrelation in ^{13}C time-series

A tree ring formed in a particular year may have had significant input of stored glucose monomers from previous years. If so, ^{13}C time-series should exhibit autocorrelation signals. To test this possibility, we correlated each ^{13}C time-series with three temporally lagged versions of itself. No significant autocorrelation (r=0.37 to 0.40 for p≤0.05 with n=28 to 25) was detected (Tables S2, S3). Therefore, our analyses does not consider conditions of previous years.

Table S2. Pearson correlation coefficients of $\Delta_i(t)$ versus $\Delta_i(t+\tau)$ and $\Delta(t)$ versus $\Delta(t+\tau)$, with t and (t+τ) denoting ^{13}C time-series without and with temporal lag, respectively.

τ [years]	Δ_1	Δ_2	Δ_3	Δ_4	Δ_5	Δ_6	Δ
-1	0.22	0.29	0.10	-0.11	-0.23	0.25	0.07
-2	0.11	0.22	0.09	0.02	-0.01	-0.10	0.09
-3	-0.04	0.00	-0.30	-0.15	-0.09	-0.11	-0.19

Table S3. Pearson correlation coefficients of $\Delta_i'(t)$ versus $\Delta_i'(t+\tau)$, with t and (t+τ) denoting ^{13}C time-series without and with temporal lag, respectively.

τ [years]	Δ_1'	Δ_2'	Δ_3'	Δ_4'	Δ_5'	Δ_6'
-1	0.23	0.33	0.12	-0.10	-0.25	0.28
-2	0.07	0.26	0.07	-0.03	-0.01	-0.24
-3	-0.05	0.10	-0.27	-0.08	0.03	-0.18

Determination of growing season length

We define the growing season as the months with a median number of days ≥10, over the observation period 1961-1995, with an average air temperature ≥5 °C. Figure S2 shows the number of days per month with ≥5 °C at Hohe Warte (Vienna, Austria, WMO ID: 1103500) during the period 1961-1995 (n=35). According to the applied definition, the growing season at our site extends from March to November.

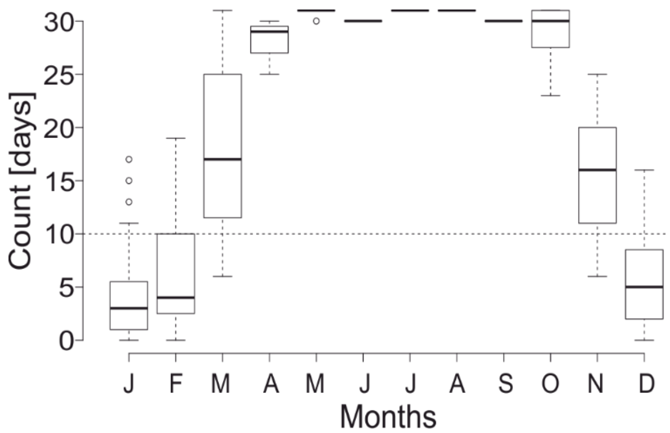

Figure S2. Number of days per month with average air temperatures ≥5°C during the period 1961-1995 at the Hohe Warte meteorological station (Vienna, Austria, WMO ID: 1103500).

Effects of air vapour pressure deficit on ^{13}C discrimination

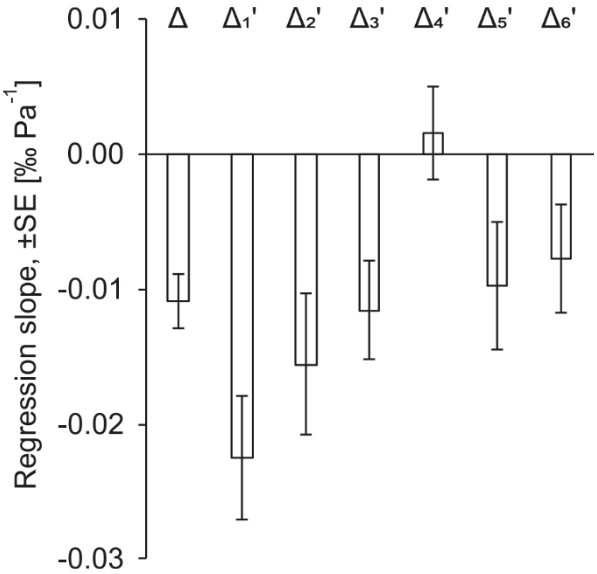

Figure S3. Effects of growing season air vapour pressure deficit (VPD) on whole-molecule ^{13}C discrimination, Δ, and on positional ^{13}C discrimination, Δ_i'. Data were acquired for tree-ring glucose of *Pinus nigra* laid down from 1961 to 1995 at a moisture-limited site in the Vienna basin. Bars represent slopes of ordinary least squares regressions, b and b_i' ±SE.

ANCOVA

Growing season air vapour pressure deficit (VPD) is significantly correlated with both Δ and Δ_1' (r=-0.72, p=5.4*10^{-6} and r=-0.68, p=3*10^{-5}, respectively, n=31). However, the slope of the Δ~VPD regression is only half as steep as the slope of the Δ_1'~VPD regression (Fig. 2, -0.011±0.002SE ‰ Pa^{-1} and -0.023±0.005SE ‰ Pa^{-1}, respectively). According to ANCOVA, this slope difference is statistically significant (p=0.02, Table S4).

Table S4. Summary of iso~VPD*method ANCOVA modelling output, with iso denoting combined Δ and Δ_1' data, VPD denoting growing season air vapour pressure deficit, and method as a categorical variable assigning iso data to either Δ or Δ_1'. Asterisks denote the significance of model terms (*, p≤0.05; ***, p≤10^{-3}). The interaction term vpd:method contributes significantly to the model (p=0.02), showing that responses of Δ and Δ_1' to VPD differ significantly.

	Sum of squares	Df	F value	Pr(>F)	Significance
**vpd**	67.315	1	45.24	8.45E-09	***
**method**	121.947	1	81.95	1.086E-12	***
**vpd:method**	8.092	1	5.44	**0.0232**	*****

References

1. Leal, S., Eamus, D., Grabner, M., Wimmer, R. & Cherubini, P. Tree rings of *Pinus nigra* from the Vienna basin region (Austria) show evidence of change in climatic sensitivity in the late 20th century. *Canadian Journal of Forest Research* **38**, 744-759 (2008).
2. Leal, S., Melvin, T.M., Grabner, M., Wimmer, R. & Briffa, K.R. Tree-ring growth variability in the Austrian Alps: the influence of site, altitude, tree species and climate. *Boreas* **36**, 426-440 (2007).

3. Strumia, G., Wimmer, R. & Grabner, M. Dendroclimatic sensitivity of *Pinus nigra* Arnold in Austria. *Dendrochronologia* **15**, 129-137 (1997).
4. Strumia, G., PhD thesis: Tree-ring based reconstruction of precipitation in Eastern Austria. (Institute of Botany, University of Natural Resources and Life Sciences, Vienna; 1999).
5. Wimmer, R., Strumia, G. & Holawe, F. Use of false rings in Austrian pine to reconstruct early growing season precipitation. *Canadian Journal of Forest Research* **30**, 1691-1697 (2000).
6. Speer, J.H. Fundamentals of tree-ring research. (The University of Arizona Press, Tucson; 2010).
7. Chaintreau, A. et al. Site-specific ^{13}C content by quantitative isotopic ^{13}C Nuclear Magnetic Resonance spectrometry: A pilot inter-laboratory study. *Analytica Chimica Acta* **788**, 108-113 (2013).
